# Breeding origins of a uniquely regular migrant songbird in the Galápagos Islands

**DOI:** 10.1002/ece3.9697

**Published:** 2023-01-16

**Authors:** Noah Perlut, Logan M. Maxwell, Adrienne Kovach, Patricia Parker, Rosalind B. Renfrew

**Affiliations:** ^1^ School of Marine and Environmental Programs University of New England Biddeford Maine USA; ^2^ Department of Natural Resources and the Environment University of New Hampshire Durham New Hampshire USA; ^3^ Department of Biology University of Missouri – St. Louis St. Louis Missouri USA; ^4^ Vermont Center for Ecostudies Norwich Vermont USA

**Keywords:** Bobolink, Charles Darwin, ddRAD, songbird

## Abstract

Little is known about the causes and consequences of alternative pathways flown by long‐distance migratory birds. Bobolinks (*Dolichonyx oryzivorus*) breed in grasslands across northern North America and migrate from their breeding grounds toward the eastern Atlantic Coast and then proceed through the Caribbean to South America. However, a small but regular number of Bobolinks have been recorded on the Galapagos Islands. We collected genetic samples from nine Galapagos Bobolinks and performed double‐digest restriction site‐associated sequencing. We compared them with samples from seven locations across their breeding distribution to determine their population of origin. Galapagos Bobolinks shared the genetic structure of a cluster in the eastern portion of the breeding range that includes New Brunswick and Ontario, Canada, and Vermont, United States. Genetic assignment tests largely corroborated this finding, although slightly different results were obtained for the two methods. All individuals were assigned to the Ontario breeding population using AssignPop, while Rubias assigned six of the migrants to Ontario and three to a Midwest breeding population. Low average relatedness among Galapagos individuals indicates that they are not more related to one another than to individuals within a breeding population and are therefore likely not from a single, small isolated population. Our results do not support the probability hypothesis—that Galapagos Bobolinks originated from the region that includes the greatest proportion of their breeding range (Great Plains)—or the vagrant hypothesis—that migrants are displaced onto Galapagos due to weather events. Instead, our findings support the proximity hypothesis, where migrants originate from the geographically closest‐breeding populations.

## Graphical abstract

Little is known about the causes and consequences of alternative pathways flown by long‐distance migratory birds. Using population genetic analyses, we compared samples from migratory Bobolinks stopping over in Galapagos with samples from seven locations across their breeding distribution to determine the migrant's breeding population of origin. Galapagos Bobolinks shared the genetic structure of a cluster in the eastern portion of the breeding range in North America that includes New Brunswick, Vermont, and Ontario.

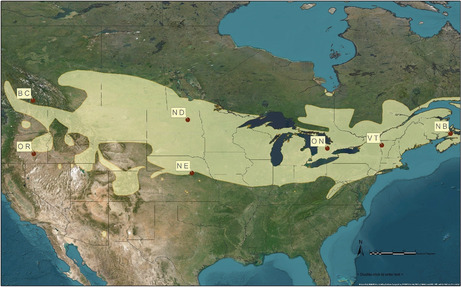

## 1 INTRODUCTION

Birds face many ecological and evolutionary trade‐offs when responding to the challenges of long‐distance migration, particularly when navigating large ecological barriers. For example, morphological traits that favor higher maneuverability can enable individuals to take a less risky but more energetically challenging route (Corman et al., 2014). Increased body size, enabling greater cold tolerance, can differentiate migration strategies between sexes (MacDonald et al., 2016). Responses can also be plastic as an individual songbird's migration strategies can differ notably between years (Fraser et al., 2019). Some birds balance the timing of their migration relative to their fat stores, exchanging the ability to expediently complete the full migration with achieving optimal timing of arrival on the breeding grounds (Prop et al., 2003). Cumulatively, these and other trade‐offs result in a complex matrix of between‐ and within‐species variation in migration strategies.

A songbird's breeding longitude and latitude can explain variation in migration routes that entail circumnavigating or crossing large barriers (Fraser et al., 2013) or lead to more time spent on migration (Neufeld et al., 2021). For example, all nightingales (*Luscinia megarhynchos*) tracked from three populations across their European breeding distribution took detours from the shortest route possible, and the detour extent varied among populations and between spring versus fall migration (Hahn et al., 2014). When crossing large barriers, birds may take the shortest path with a tailwind, rather than wait for optimal wind conditions that would enable a longer non‐stop flight (Abdulle & Fraser, 2018). Individuals who are capable of crossing a large barrier without stopping may experience an advantage by minimizing total migration time (Bennett et al., 2019) and reducing the overall energy costs of migration (Schmaljohann et al., 2010).

The Bobolink (*Dolichonyx oryzivorus*), a grassland obligate songbird in the blackbird family (Icteridae), migrates between North and South America each year. Its breeding range spans nearly coast to coast in Canada and the United States. Populations from British Columbia to Nova Scotia migrate through the Caribbean and the Llanos of Venezuela and Colombia to and from their wintering grounds in Bolivia and Argentina (Renfrew et al., 2013). There is at least one consistent exception to their general migratory route. Beginning with Charles Darwin in 1835 (Darwin, 1963), observations of small numbers of Bobolinks on the Galápagos Islands have been documented on numerous occasions, including observations in at least 13 different years between 1957 and 2021 (Table 1). Numbers of birds and observations are greatest during October and November, and generally higher during fall and spring than any other time of the year, although individuals have been observed on the islands in most months of the year (Lévêque et al., 1966). Harris (1973) suggested that numbers of individuals “vary greatly from year to year,” although systematic surveys have never been conducted.

**TABLE 1:** Records of Bobolinks on the Galápagos Islands.

Date	Island	No. Bobolinks	Observer(s)	Source
Sept 1836	San Cristóbal (Chatham)	1	Charles Darwin	Darwin (1963)
	Charles, San Cristóbal (Chatham)			Rothschild and Hartert (1899)
Aug, Sept 1957	Santa Cruz	Several adults	Bowman	Wiedenfeld (2006)
Oct 16, 1961	Santa Cruz (Indefatigable)	2	R. Leveque	Lévêque et al. (1966)
Nov 21, 1962	Santa Cruz (Indefatigable)	1 collected	Brosset	Lévêque et al. (1966)
July 25, 1963	Genovesa (Tower)	1	Galápagos Historical Data	eBird (2019)
Jul 25, 1963	Genovesa	1 emaciated male collected	P. Kramer	Kramer (1965)
Oct 15, 1964	Española	1	Not provided	Lévêque et al. (1966)
Oct 22, 1964	Santa Cruz (Indefatigable)	1	Miguel Castro	Lévêque et al. (1966)
Oct 27, 1964	Santa Cruz (Indefatigable)	10	Miguel Castro	Lévêque et al. (1966)
Nov 11, 1964	Santa Cruz (Indefatigable)	1	Miguel Castro	Lévêque et al. (1966)
Dec 13, 1964	Santa Cruz (Indefatigable)	1	Miguel Castro	Lévêque et al. (1966)
circa 1967	Santa Cruz (Indefatigable)	many flocks of up to 20 individuals	Alf Kastdalen	Lévêque et al. (1966)
Aug 11, 1967	Santa Cruz (Indefatigable)	1 dead (decomposed)	Alf Kastdalen	Lévêque et al. (1966)
Nov 20, 1970	Marchena	1	skin at MECCD	Wiedenfeld (2006)
Nov 26, 1978	Gardner‐by‐Española	1	skeleton at MECCD	Wiedenfeld (2006)
Apr 27, 1980	Española	1	Gayle Davis; Harris pers. comm.	Wiedenfeld (2006)
Sept 27, 1980	Pinta	1	Harris pers. comm.	Wiedenfeld (2006)
May 29, 1983	Genovesa (Tower)	1	Scott Stoleson	eBird (2019)
Nov 17, 1983	Española	1	Mark Van Beirs	Wiedenfeld (2006)
Dec 9, 1983	Española	1	Mark Van Beirs	Wiedenfeld (2006)
Nov 18, 1996	Santa Cruz (Indefatigable)	3	Michael Dvorak	eBird (2019)
Apr 22, 2004	Española	2	Galápagos Historical Data	Wiedenfeld (2006)
Dec 8, 2009	San Cristóbal (Chatham)	1	Ian Gardner	eBird (2019)
Nov 6, 2013	Española	1	Steve Holliday	eBird (2019)
No date given	Floreana (Charles)	specimen(s) collected	Not provided	Lévêque et al. (1966)
No date given	Santiago (James)	specimen(s) collected	Not provided	Lévêque et al. (1966)
Oct 12–232015	San Cristóbal (Chatham)	9 captured, ~25 observed	N. Perlut, J. Megyesi, R. Renfrew	Perlut and Renfrew (2016)
Oct 5–312021	San Cristóbal	6 captured, ~20 observed	N. Perlut	N. Perlut (unpublished data)

*Note*: Historical island names used in older records follow contemporary names, in parentheses.

Abbreviation: MECCD, Museo de Vertebrados e Invertebrados Marinos de la Estación Científica Charles Darwin.

The breeding origins, migratory routes, and non‐breeding locations of Bobolinks that stop in Galapagos are unknown. Pettingill Jr. (1983) hypothesized that they breed west of the Continental Divide, migrate south through western Mexico, and continue across the Pacific Ocean. Bobolinks breeding in Oregon and British Colombia, however, have so far been shown to use the species' main migratory route by flying east from their breeding grounds toward the East Coast and then proceeding through the Caribbean to northern South America (Renfrew et al., 2013, 2020). Thus, to date, we lack an understanding of the migratory pathways, including the breeding grounds of origin, for the birds that stopover on Galapagos.

Identifying the breeding origins of Bobolinks stopping in the Galapagos Islands will not only contribute to our understanding of avian migration patterns but it is also important to the conservation of resident bird species on the islands. Bobolink is the only songbird that regularly migrates through the Galapagos Islands, and it carries at least two Plasmodium lineages that infect endemic Galapagos birds (Levin et al., 2013), presumably through the vector of two recently introduced species of mosquitoes. Based on a database of parasites in co‐occurring grassland bird species, one of these lineages is likely obtained by Bobolink on migration stopover, while the other is more likely transmitted to Bobolink on its breeding grounds (Levin et al., 2016). However, although four of nine Bobolinks sampled in the Galapagos Islands during their southbound migration carried Plasmodium, the lineages were not those previously identified in Galapagos endemic birds; these infections provide a catalog with which to monitor for future parasite infections (Perlut et al., 2018). Elucidating the breeding origin(s) of Galapagos Bobolinks, therefore, may shed light on transmission routes of the various Plasmodium lineages, including those affecting endemic birds in Galapagos.

We propose four main hypotheses, not mutually exclusive, to explain why some Bobolinks migrate through Galapagos. (1) Under the *probability hypothesis*, by chance, Galapagos birds are from a part of the range where the majority of the global population breed. Here, we expect that Galapagos‐bound Bobolinks would originate from the Great Plains region. (2) The *vagrant hypothesis* suggests that Bobolinks are in Galapagos as a result of weather events that have displaced them from their usual migration route. Under this hypothesis, we expect that Bobolinks that stop on Galapagos would originate from multiple breeding populations. This hypothesis could also be supported if Galapagos Bobolinks originate from only one region, and by chance, the weather events affect this region only. (3) The *proximity hypothesis* predicts that Galapagos birds originate from the part of the breeding range that is the shortest orthodromic distance from Galápagos. Under this hypothesis, we expect that Galápagos Bobolinks would originate from the southern part of midwestern or eastern breeding populations. (4) Under the *alternate non‐breeding range hypothesis*, Bobolinks stopping in the Galápagos Islands are passing through on their way to spend the austral summer in Ecuador, potentially on the islands themselves, in Ecuador west of the Andes, or in Peru (areas outside of their main wintering range).

Here, we use genetic analyses to examine the breeding origins of Bobolinks that stop in Galápagos during their southbound migration. We assign individuals to their breeding region based on the known genetic population structure of Bobolink across its breeding range (Renfrew et al., 2021) using two genetic assignment methods, and we assess whether they originated from a single, local breeding area by determining their genetic relatedness.

## 2 METHODS

### 2.1 Sample collection and preparation

On October 12–23, 2015, we searched for Bobolinks on migration stopover in native and agricultural grasslands in the highlands of San Cristóbal Island, Galápagos, Ecuador (Perlut & Renfrew, 2016). We used mist nets and playbacks to capture nine Bobolinks at Finca de las Gemelas (422 m elevation; 0°53′26.23″S, 89°27′15.35″W), a 2.5 ha grassland patch, and in a pasture at Santa Monica, an agricultural complex 9.15 km west of Gemelas, owned by the Ecuadorian military (435 m elevation). We fitted each bird with a unique U.S. Geological Survey leg band, recorded morphometric measurements, and collected a blood sample from the brachial vein into lysis buffer for DNA preservation (Longmire et al., 1988). We used a standard phenol–chloroform extraction protocol following Sambrook et al. (1989), with a final dialysis step in TNE2 (1 M Tris pH 8, 5 M NaCl, 0.5 M EDTA, and dH2O), for DNA extraction of the Bobolink samples. Using a Qubit fluorometer broad‐range double‐stranded DNA assay kit (Life Technologies), we determined the DNA concentration of each sample. Samples below 10 ng/μl were concentrated through vacuum centrifuge, while samples above 25 ng/μl were diluted, resulting in a concentration range 5–25 ng/μl across samples.

### 2.2 Molecular and bioinformatic analyses

**FIGURE 1:**
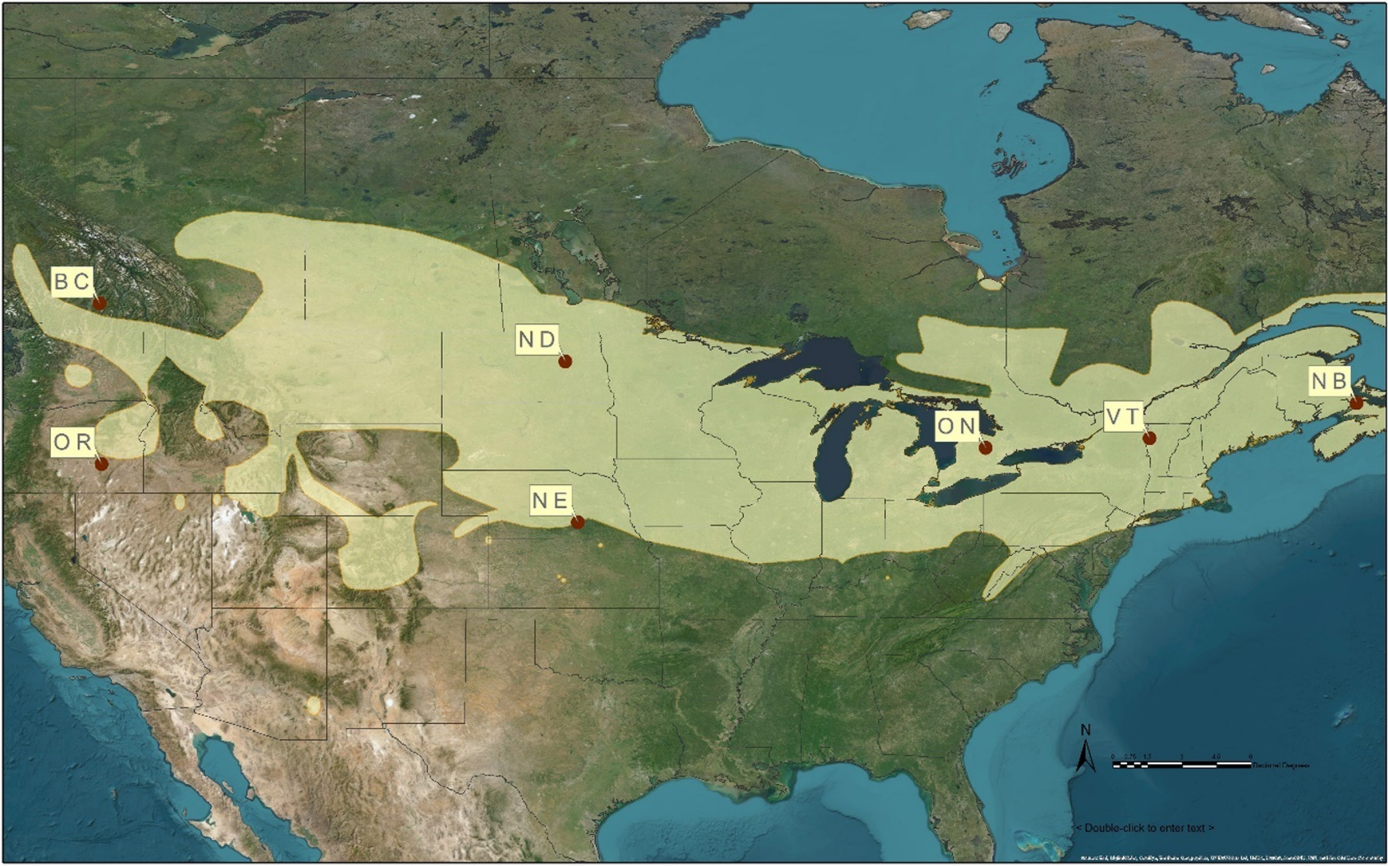
Our seven sampling locations, including New Brunswick (NB), Vermont (VT), Ontario (ON), North Dakota (ND), Nebraska (NE), British Columbia (BC), and Oregon (OR). The Bobolink breeding distribution is represented in tan.

The nine Galápagos samples presented here were sequenced in conjunction with 174 samples collected from seven populations (Figure 1) in the Bobolink breeding range, as part of a study on population structure (Renfrew et al., 2021). Analyzing these datasets together (total of 183 individuals) allowed for determining breeding population of origin for the birds caught in Galápagos. The seven breeding sampling locations of origin—Ontario, New Brunswick, Vermont, North Dakota, Nebraska, Oregon, and British Columbia—were structured into four genetically distinct regions/populations—East (Ontario, New Brunswick, and Vermont), Midwest (North Dakota and Nebraska), Oregon, and British Columbia (Renfrew et al., 2021).

We created double‐digest restriction‐site‐associated (ddRAD) sequencing libraries for the nine Galápagos samples in this study and the 174 Bobolink breeding population samples, following the Peterson et al. (2012) protocol, as described in Renfrew et al. (2021). Briefly, we digested 200–500 ng of DNA with the enzymes SbfI and MsPI, followed by ligation of P1/P2 adapters using T4 DNA ligase. Samples were pooled into index groups using unique P1 adapters (Peterson et al., 2012) and cleaned using 1.5× Agencourt AMPure XP beads. BluePippin (Sage Science) was used to select fragments between 400 and 700 bp and Illumina TruSeq primer sequences were incorporated into the libraries using a low‐cycle PCR. We performed a final clean‐up using AMPure XP beads, and the resulting libraries were visualized on a fragment bioanalyzer to ensure correct pooling, fragment size, and distribution. Final libraries were sequenced at the Cornell University Institute for Biotechnology Genomes Facility Research Center, in a single Illumina HiSeq 2500 rapid run lane (100 bp reads).

Bioinformatic data processing and SNP identification are also described in Renfrew et al. (2021). In summary, we checked all raw DNA sequences for quality using FastQC (Andrews, 2010). Using the FASTX‐Toolkit (Hannon, 2010), we trimmed all sequences on their 3′ end to 97 bp and eliminated reads that had a Phred quality score ≤10, in addition to 95% of the reads that scored <20. Using process_radtags in STACKS (version 2.0), we demultiplexed all reads, while also filtering for low‐quality sequences, those that did not meet the Illumina chastity/purity filter, had an uncalled base, or had one or more adapter mismatches. We further required that reads had an intact SfbI RAD cut site and one unique barcode. The resulting reads were trimmed using fastx_trimmer to the length of the shortest sequence (90 bp).

We used the STACKS (version 2.0) pipeline to identify SNPs from these filtered sequences. We optimized parameters for the de novo assembly, as described in Renfrew et al. (2021), resulting in *m* = 3 (number of identical reads required to initiate a new putative allele) and *M* and *n* = 5 (number of mismatches allowed between stacks within individuals and number of mismatches allowed between stacks between individuals). We used program Populations in STACKS to group individuals into eight geographic sampling locations (seven breeding locations and Galápagos), from which we identified polymorphic SNPs. We required an SNP to be present in 70% of individuals and six populations to be called. We also required a minimum minor allele frequency of 0.01 and maximum observed heterozygosity of 0.5 to process a nucleotide site at a locus. Only the first SNP per stack was used to avoid linkage in the dataset. This resulted in a final SNP panel of 3234 SNPs. We tested for conformance to Hardy–Weinberg equilibrium and validated that missing data in our final dataset had no effect on inference of population structure (Renfrew et al., 2021). We also used the program BayeScan (version 2.1; Foll & Gaggiotti, 2008) to identify loci putatively under selection within our dataset, and we removed eight loci that were identified as outliers, to form a putatively neutral SNP panel (3226 loci).

### 2.3 Population‐level analyses and relatedness

To characterize genetic structure of the Galápagos migrants in relation to the four previously identified breeding populations (Renfrew et al., 2021), we used a Bayesian clustering algorithm in program STRUCTURE (version 2.3.4; Pritchard et al., 2000). Using our neutral SNP panel, we ran 10 runs of 150,000 MCMC repetitions with a burn‐in of 50,000 repetitions for each *K* value (*K* = 1–8). We used the LocPrior model (Hubisz et al., 2009) with correlated allele frequencies. We determined the allele frequency parameter used in subsequent analyses by performing an initial run of *K* = 1, allowing lambda to vary and settle at the correct value (lambda = 0.37). To assess the results of STRUCTURE and determine the best‐supported model, we used a combination of the delta *K* method (Evano et al., 2005) and plateau in lnPD criterion (Pritchard et al., 2000) from program Structure Harvester (Earl & vonHoldt, 2012). We visualized results using CLUMPAK (Kopelman et al., 2015).

We performed a discriminate analysis of principle components (DAPC) with the Adegenet package in program R as an additional method to assess genetic differentiation among the samples (Jombart, 2008) and determine with which breeding population(s) Galápagos migrants cluster most closely. We first transformed the data using a principal components analysis and then determined genetic clusters using discriminant analysis. We ran this analysis with our neutral SNP panel (3226) with inferred populations as our sampling locations. We performed a cross‐validation to accurately determine the number of principal components to retain (78 PCs and seven discriminant functions retained).

To test whether Galápagos birds likely originated from a single breeding locale or had more diverse population origins, we evaluated population‐level inbreeding coefficients and relatedness values, in comparison with the birds from the seven breeding locations. We hypothesized that if Galápagos birds had higher than average population‐level relatedness and inbreeding values, this would provide evidence consistent with originating from a single locale. We calculated inbreeding coefficients (*F*_is_ values) for each of the seven breeding locations and the nine Galápagos migrants, in the program Populations of STACKS. We calculated pairwise individual relatedness values for each bird using ‐‐realtedness2 function in VCF tools (Danecek et al., 2011) following the method described in Manichaikul et al. (2010), and averaged them across sampling locations.

### 2.4 Assignment tests

We used two methods of genetic assignment testing to determine the origin of Galápagos migrants to their breeding population—AssignPop (Chen et al., 2018) and Rubias (Moran & Anderson, 2018), both implemented in program R (R Core Team). Both methods employ a framework that is designed for solving the upward bias issue previously documented with these types of analyses (Anderson, 2010; Waples & Do, 2010) but differs in their algorithms and analytical approach. Since different population genetic methods may lead to slightly different results, we followed standard practice of employing more than one method.

AssignPop uses a supervised machine‐learning method to evaluate discriminatory power of a dataset to correctly assign individuals of unknown origins to source populations (Chen et al., 2018). It uses principal component analysis for dimensionality reduction, Monte–Carlo resampling cross‐validation to estimate mean and variance in assignment accuracy, and machine‐learning classification algorithms to build predictive models. We used individuals from our seven breeding population sampling locations as a training dataset to subsequently assign Galápagos birds back to their breeding population of origin. Because outlier loci may improve assignment testing power, and our prior work confirmed that the population structure recovered by outliers does not differ from that of neutral loci (Renfrew et al., 2021), we performed this analysis with all SNP loci. To confirm the outlier loci did not provide any assignment bias, we also ran the assignment testing using the neutral SNP panel. We used the Support Vector Machine (SVM) model (30 iterations) with all loci for the cross‐validation while varying the number of individuals (50%, 70%, and 90%). To ensure that unequal sample sizes were not influencing assignment accuracy, we also ran the validation with varying numbers of individuals (4, 6, and 12 individuals) for comparison. Once accuracies were calculated on the training dataset, we ran the assignment test using the naive Bayes model to determine the breeding population of origin for the migrant Galápagos Bobolinks.

Rubais employs an approach that has traditionally been used in genetic stock identification (GSI) in fisheries, and it is powerful for estimating mixture proportions and individual assignments in other systems as well (Moran & Anderson, 2018). It uses a Bayesian inference in the conditional GSI model and employs a leave‐one‐out cross‐validation method for accurate prediction of model accuracy. Through cross‐validation and simulation, reporting unit bias can be explored within the reference data, and a parametric bootstrap approach can be employed in the subsequent mixture analysis to correct for bias, if appropriate. Our reference dataset for this analysis included the 174 individuals from the seven breeding sampling locations (comprising seven reporting units), and our mixture (unknown individuals for assignment) included the nine migrant Bobolinks captured in Galápagos. Because AssignPop results with the full SNP panel (including outliers) provided consistent and slightly better results than the neutral SNP panel (see Section 3), we ran this analysis with the full SNP panel.

First, we assessed the power of the reference dataset using the self‐assign function, by which reference individuals were assigned back to sampling collections using a leave‐one‐out procedure, as summarized in Anderson et al. (2008). We then evaluated assignment accuracy through simulated mixtures, using the same leave‐one‐out approach. We estimated mixing proportions of simulated individuals using a maximum‐likelihood EM algorithm and posterior mean from MCMC. We ran these simulations with a maximum size of 50 individuals in each simulated mixture, with 100 MCMC repetitions. Simulations were carried out using full multilocus genotypes, as well as resampling over gene copies, as the latter method was found to yield a better power assessment in some instances (Anderson et al., 2008). We compared estimated reporting unit mixing proportions to simulated values to assess the degree and direction of reporting unit bias within the data. Subsequently, we ran a mixture analysis on the nine Galápagos birds using both the default MCMC method as well as the parametric bootstrapping method to account for reporting unit bias found in our data. We ran the above procedure with reporting units defined at both a regional (four genetically distinct populations of Renfrew et al., 2021) and local scale (seven breeding sampling locations) to determine the breeding population of origin of the birds in the mixture.

## 3 RESULTS

We had a total of 111,771,061 raw sequencing reads, of which we retained 72,554,594 after initial data quality filtering (average of 356,316 reads per individual). After de novo assembly (optimized and summarized in Renfrew et al. (2021)), we were left with a stack catalog of 71,370 loci. For the full panel of 3234 SNPs, there was a mean sequencing depth of 41× across all individuals (SD = 32).

**FIGURE 2:**
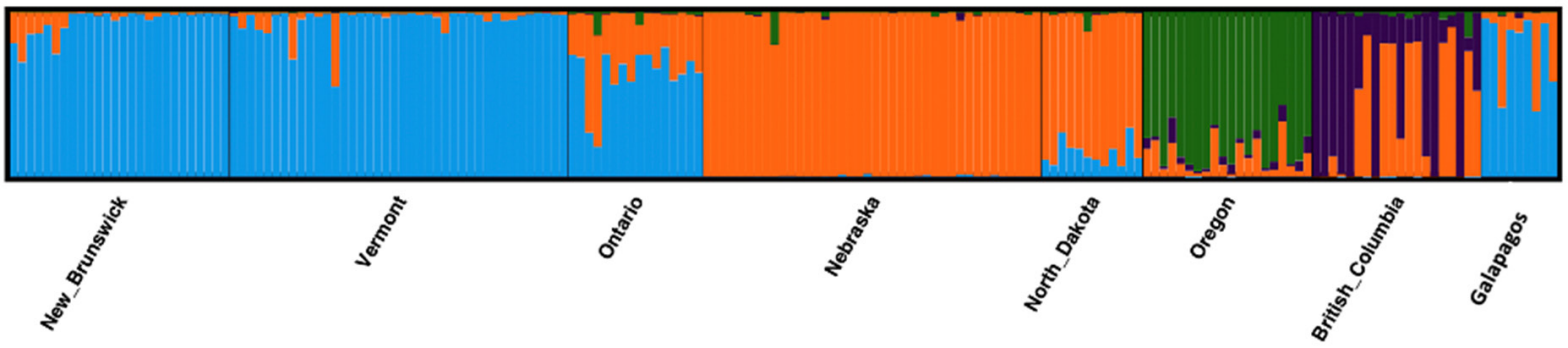
Bar plot from STRUCTURE analysis of Bobolink breeding sampling locations and migrants caught on San Cristobal Island, Galápagos, at *K* = 4 population structure identified in previous studies (3226 SNPs). Each vertical bar shows individual membership to each genetic cluster (shown here in east–west geographical manner).

Using the delta *K* method, STRUCTURE analysis supported a *k* = 3 solution; however, the plateau in the estimated log probability of the data (LnP(D)) suggested a model of *k* = 4 (Figure 2; Appendices S1 and S2). The bar plots further confirmed a solution at *k* = 4, consistent with the population structure found in our previous study of Bobolink population structure: Vermont and New Brunswick formed an eastern population, Nebraska and North Dakota formed a midwestern cluster, with Ontario showing admixture between the eastern and midwestern populations, and Oregon and British Columbia each had their own unique genetic signal with some gene flow with the midwestern cluster (Figure 2). The migrant Galápagos birds clustered with the eastern population, although three individuals show mixed ancestry with the mid‐west cluster, similar to the pattern seen in birds in the Ontario population (Figure 2).

**FIGURE 3:**
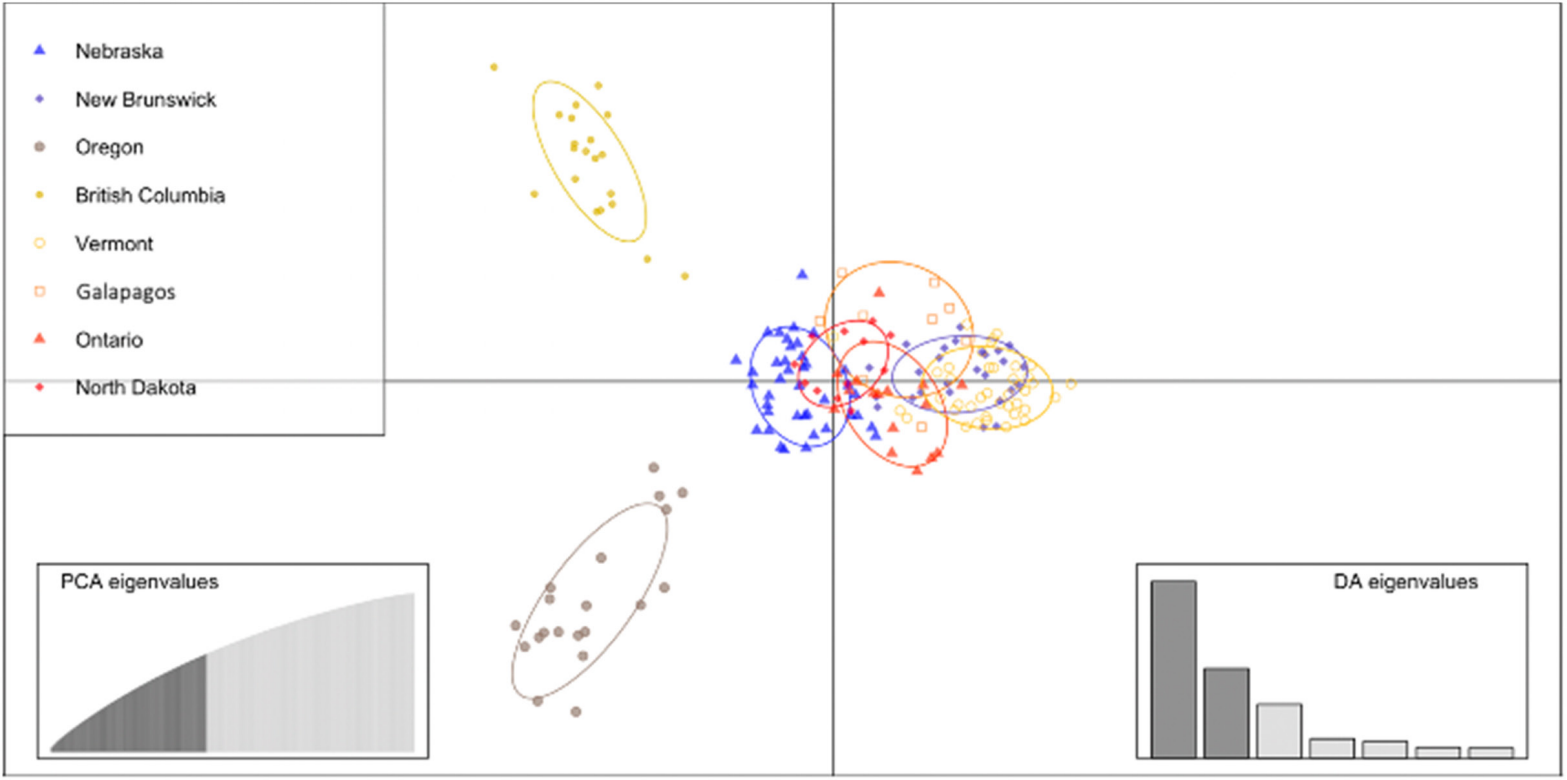
Discriminant analysis of principal components (DAPC) scatter plot with the seven breeding sampling locations of Bobolinks and the migrants caught on San Cristobal Island, Galápagos. Each dot represents an individual with each color denoting where the samples were collected in 95% inertia ellipses. Insets show DA and PCA eigenvalues. A total of 78 PCSs and 7 discriminant functions were retained, and 61.3% of variation was explained.

Discriminate analysis of principle components mirrored results from the STRUCTURE analysis (Figure 3). Oregon and British Columbia cluster in their own unique genetic spaces in both the longitudinal and vertical axes. Vermont and New Brunswick (eastern), as well as North Dakota and Nebraska (midwestern), cluster closely together and have considerable overlap in their 95% inertia ellipses. The midwestern and eastern clusters separate along the longitudinal axis, with the Ontario individuals and the Galápagos migrants spanning the space between the midwestern and eastern populations, although they appear to overlap more with the eastern cluster than the midwestern cluster.

Inbreeding coefficients (*F*_is_) were similar among all the sampling locations (range 0.083–0.147; Table 2), with levels typical of outbred populations. The Galápagos migrants had the lowest *F*_is_ value (0.0830), while Nebraska exhibited the highest value (0.147). Mean pairwise relatedness within populations was low for all sample locations (range −0.1835 to −0.2739; Table 2), indicating sampled individuals were unrelated. Although all values were similarly low, the Galápagos individuals had the lowest average pairwise relatedness value when compared to the breeding populations, illustrating that they are no more related to one another than those found in any of the breeding populations sampled (Table 2).

**TABLE 2:** Inbreeding coefficients (*F*_is_) and mean pairwise relatedness for each of the seven breeding sampling locations of Bobolinks, along with the migrants caught on the Galápagos Island.

Population	Average relatedness	Inbreeding coefficient (*F*_is_)
Nebraska	−0.2408	0.1469
New Brunswick	−0.2185	0.1175
Oregon	−0.1962	0.0839
British Columbia	−0.2305	0.0879
Vermont	−0.2376	0.1442
Galápagos	−0.2739	0.0830
Ontario	−0.1825	0.0873
North Dakota	−0.2377	0.0840

**FIGURE 4:**
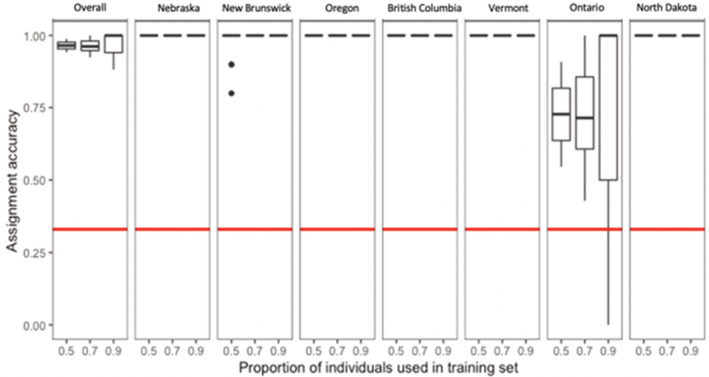
Results of the Monte–Carlo cross‐validation performed in AssignPop to evaluate the baseline Bobolink data (reference dataset of 174 individuals from seven breeding locations). Assignment accuracy was estimated through resampling random training individuals using all loci (3234 SNPs). Each inset shows assignment accuracy in the baseline data across each sampling location and overall at varying proportions of individuals used in the training dataset (0.5, 0.7, and 0.90).

Monte–Carlo cross‐validation of the baseline data in AssignPop resulted in high assignment accuracies overall and for every sampling location across varying proportions of individuals used in the training dataset, with the full 3234 SNP panel (Figure 4). Mean assignment accuracies were 100% for Nebraska, New Brunswick, Oregon, British Columbia, Vermont, and North Dakota at each proportion level (0.5, 0.7, and 0.9), while Ontario had slightly lower levels at each proportion level (73%–100%; Figure 4). Because we had high confidence in the power of the baseline dataset for individual assignments, we ran the assignment test in AssignPop to assign the nine Galápagos migrants back to one of the seven breeding sampling locations. The model assigned each of the migrant birds to the Ontario population with high confidence (>0.99 assignment; Table 3). Similar results were found when the analysis was performed using neutral loci (3226 SNPs) only, although with slightly lower assignment scores (Appendix S3).

**TABLE 3:** Results from AssignPop assignments of nine migrant Bobolinks caught on the Galápagos Islands back to breeding locations using all loci (3234 SNPs).

Individual	% Assignment to Ontario
Galápagos 44619	0.999
Galápagos 44620	0.999
Galápagos 44621	0.999
Galápagos 44622	0.999
Galápagos 44623	0.999
Galápagos 44624	0.999
Galápagos 44625	0.999
Galápagos 44626	0.999
Galápagos 44627	0.998

*Note*: All nine individuals were assigned to the Ontario breeding population with high confidence.

In Rubias, the self‐assignment testing of reference individuals resulted in high average posterior means of group membership (P of Z) for the four regional breeding population groups (eastern, midwestern, Oregon, and British Columbia), with all groups having >89% correct assignment (Table 4). Self‐assignment testing also resulted in high P of Z values for six of the seven breeding sampling locations (range 90%–100%), apart from North Dakota which had an average posterior means of group membership of 40% (Table 4).

**TABLE 4:** Results of evaluation from the Self Assign function in Rubias, for the 174 Bobolink reference individuals from the seven breeding locations (left) and the four regional breeding groups (right).

7 Breeding Loc.	Self‐assignment	Simulated mixture results			4 Regional populations	Self‐assignment	Simulated mixture results	
	Average P of Z	Mixture proportion (Pi)	Bootstrapped correction			Average P of Z	Mixture proportion (Pi)	Bootstrapped correction
Vermont	99.999	0.025	0.019	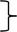	East	98.665	0.644	0.979
New Brunswick	80.409	0.013	0.006					
Ontario	90.469	0.606	0.626					
North Dakota	39.855	0.013	0.000	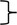	Midwest	99.999	0.327	0.000
Nebraska	100.000	0.315	0.332					
Oregon	100.000	0.015	0.008		Oregon	100.000	0.014	0.001
British Columbia	89.962	0.015	0.008		British Columbia	89.963	0.0148	0.011

*Note*: The average posterior means of group membership (P of Z) show the average posterior probability of assignment to the correct group for each reporting unit. Simulated mixture proportions (pi) and parametric bootstrapped corrections on those mixture proportions for the migrant Galápagos Bobolink mixture dataset are shown after the self‐assignment results, calculated in program Rubias for the seven breeding locations (left) and the four regional population groups (right).

For the simulated mixtures in Rubias, the average P of Z was consistently high across regional breeding population simulations (eastern, midwestern, Oregon, and British Columbia), regardless of the method of resampling, ranging from 99% to 100% with the gene copy method and 91%–100% with the full multilocus method (Appendices S5 and S6). Average P of Z levels were also high (range 93%–100%) for simulations performed with the seven sampling locations as reporting units using the gene copy method (Appendix S4). These levels were lower and more similar to the self‐assignment results described above when the simulations were performed with the seven breeding sampling locations and the full multilocus genotype resampling (range 82%–100%, except for North Dakota at 34%; Appendix S5). We compared estimated reporting unit mixing proportions to simulated values to assess the degree and direction of reporting unit bias within the data. Plotted values showed similar patterns across all the above simulations, with differences in the magnitude of the pattern. Overall, most values followed a linear pattern, with some amount of upward bias seen in Nebraska or Midwest reporting units (Appendices S4–S7). Due to some observed evidence of bias, we subsequently performed the mixture analysis of the Galápagos birds with the parametric bootstrapping correction.

Mixture analysis in Rubias assigned the nine Galápagos individuals back to the four regional breeding populations, as well as the seven breeding sampling locations with high levels of accuracy, using both the default method and parametric bootstrapping correction for reporting unit bias. Six Galápagos migrants were assigned to the eastern cluster and three individuals were assigned to the midwestern cluster with 100% certainty (Table 5), using the bootstrapping correction. When we used the seven sampling locations as reporting units, we were able to get better resolution on breeding origin of the birds with similarly high levels of certainty (>91%). We found the same six birds that were assigned to the eastern group were assigned to Ontario and the same three individuals that were assigned to the midwestern group were assigned to Nebraska (Table 5). Assignments were identical when we ran the analysis without the bootstrapping correction, with >92% average P of Z (Appendix Table S1).

**TABLE 5:** Results of the mixture analysis of nine migrant Galápagos Bobolinks from program Rubias.

Individual	7 breeding locations rep unit	P of Z	4 regional pops rep unit	P of Z
Galápagos 44619	Ontario	1.000	East	1.000
Galápagos 44620	Ontario	1.000	East	1.000
Galápagos 44621	Nebraska	0.999	Midwest	0.999
Galápagos 44622	Ontario	1.000	East	1.000
Galápagos 44623	Ontario	0.912	East	0.999
Galápagos 44624	Ontario	1.000	East	1.000
Galápagos 44625	Nebraska	0.999	Midwest	0.999
Galápagos 44626	Ontario	1.000	East	1.000
Galápagos 44627	Nebraska	1.000	Midwest	1.000

*Note*: The table includes each individual caught on the Galápagos Islands, the reporting unit to which it was assigned (from one of the seven breeding locations or one of the four regional groups), and the associated probability of assignment (P of Z).

Mixture proportions (pi) for the analysis with the seven breeding populations matched the results of the assignment testing, with approximately 0.606 proportional assignment to Ontario, 0.315 to Nebraska, and 0–0.019 to the other five populations. Bootstrap corrected

proportions were largely similar to the uncorrected mixture proportions, albeit slightly higher to Nebraska and Ontario, thereby further strengthening the evidence for these assignments (Table 4). Uncorrected and corrected mixture proportions differed, however, for the results with the four regional breeding populations. Uncorrected mixture proportions were 0.644 to the East and 0.327 to the Midwest, consistent with the mixture analysis of the seven populations. With the bootstrapped correction, however, mixing proportions to the East were 0.979 and 0–0.011 to the other three groups, suggesting stronger evidence for assignments to the East (Table 4).

## 4 DISCUSSION

Using genetic data in conjunction with assignment testing and mixture analysis, we identified the breeding population of origin of individual Bobolinks that use an alternate migratory pathway through the Galápagos Islands. Comparing the genotypes at 3234 SNPs of nine Bobolinks captured in Galápagos during their southbound migration to population genetic data for seven locations in the Bobolink breeding range, we determined that the Galápagos migrants were genetically most similar to Bobolinks from the eastern portion of the breeding range. The two different assignment testing approaches that we used gave largely similar results, however, there were slight discrepancies. The AssignPop method assigned all nine migrants to the Ontario location within the eastern breeding population with high confidence. Rubias assigned six of the migrants to the Ontario population with high confidence and three to Nebraska (in the midwestern breeding group). When mixture analysis was conducted with the four genetically distinct breeding groups, rather than all seven breeding locations, the bootstrap corrected mixing proportions corroborated the AssignPop results, with nearly the full mixture assigned to the eastern population. Taken together, these results best support the eastern breeding population as the origin of most or all of the Galápagos migrants, although it cannot be ruled out that some individuals may have originated in the Midwest.

The slight uncertainty in the assignment testing with the Rubias approach is not surprising, given that there is admixture between the eastern and midwestern populations, particularly in Ontario (Figure 2; Renfrew et al., 2021). The three migrant individuals that Rubias assigned to Nebraska have the highest mixed ancestry in the structure bar plots (Figure 2). Rubias may have difficulty correctly assigning these individuals given the signal of admixture. The underlying GSI model used in Rubias does not handle admixed individuals; the model assumes individuals are purely from one reference group or another (Moran & Anderson, 2018). The dimensionality reduction in the PCA‐based method in AssignPop may deal with admixture in the reference dataset differently, allowing for clearer assignments in the presence of admixture. As such, and given the strong evidence of an eastern origin from the bootstrap corrected mixture proportions, we conclude that the most likely origin of the majority (if not all) of the Galápagos migrants is the eastern breeding group, and specifically, the Ontario area. Furthermore, the relatively small *F*_*is*_ and negative relatedness values for the Galápagos Bobolinks (Table 2) suggest that they are not from a single, closely related breeding population. This indicates that it is unlikely that the Galápagos migrants are from a small, isolated eastern population and may instead stem from several locations within and around the Ontario area.

In trying to explain why a small number of Bobolinks maintain a historical migration through Galápagos, our results do not support the probability hypothesis, which predicts that they originate from the region that includes the greatest proportion of the breeding population. The core of the Bobolink breeding range with the highest abundances lies in the Great Plains, particularly in North Dakota, which has a different genetic population structure from the eastern cluster (Renfrew et al., 2021). While our results could not completely rule out the Midwest as the origin of three of the migrants, these assignments were to Nebraska and not North Dakota, thereby also inconsistent with the core portion of the breeding range.

Our results also do not support the vagrant hypothesis. If Bobolinks in Galápagos were vagrants, they would be present as a result of weather or other stochastic events that alter their normal migratory route. As vagrants, we would expect the Bobolink population on the islands to comprise a mix of individuals from different locations in the breeding range. If samples collected from individuals across multiple years all originated from the eastern breeding range, this hypothesis may be ruled out, unless there is an annual weather event that always causes a detour for a few eastern individuals only. Reporting on an 1898–1899 Galápagos expedition, Snodgrass and Heller (1903) wrote in a brief account: “five hundred and fifty miles is a long distance for [the Bobolink] to traverse accidentally.” While individual vagrants do occasionally appear at such distances from their normal route—even on different continents—this phenomenon does not occur for several to dozens of individuals on a regular basis (aside from the data summarized in Table 1, there is no estimate of how many Bobolinks stopover on Galápagos each year). Bobolinks are occasional September to November vagrants in Ireland (<5 records) and the United Kingdom (<40 records; Swash et al., 2020); given this rarity and inconsistency, their vagrancy in the United Kingdom is likely explained by weather—unusual wind events pushing individual birds east. However, vagrancy can also be a byproduct of an increasing breeding population (Zawadzki et al., 2019). While Bobolink populations have declined steadily since at least the 1960s (Sauer et al., 2020), we cannot rule out that this migration pattern through the Galápagos Islands began during a period of rapid agricultural expansion in eastern North America during the 19th century, which led to rapid growth of the existent eastern Bobolink population (Askins, 1999).

Our findings may support the proximity hypothesis under at least two different scenarios; validation of either scenario requires additional data and analyses. First, the longitude of Galápagos (~−91°) is the same as the longitude where the Mississippi River divides southern Wisconsin and southern Minnesota, approximately 4900 km from the Galápagos Islands. The Mississippi River basin would therefore be the location that is the shortest orthodromic distance from the Galápagos Islands. If this region of the Midwest is genetically similar to the eastern breeding population (particularly Ontario), it would provide evidence supporting the origin of Galápagos Bobolinks in the Mississippi River basin, thereby supporting the proximity hypothesis. Alternatively, the proximity hypothesis would also be supported if the Galápagos Bobolinks originated from the southeastern portion of the breeding range (from which we also currently lack genetic data), although the Bobolinks would have to fly a portion of that route along a declination counter to that of the main route through the Caribbean. Under the vagrant hypothesis, individuals from the eastern part of their breeding distribution could have been forced west by weather events prior to southbound migration and did not correct their migratory declination accordingly. There is support for such a lack of correction: a Bobolink breeding in North Dakota that was experimentally displaced to California prior to migration did not adjust its migration declination, and instead flapped in the direction of the coast of California, toward the Pacific Ocean (Hamilton III, 1962). It is feasible that Bobolinks from the southeast of the breeding range could encounter the Galápagos Islands if they were influenced by a weather event and failed to self‐correct. Nonetheless, there is a low likelihood that this happens on a regular basis, and only to populations from one part of the breeding range.

Our study did not test the alternate non‐breeding range hypothesis, whereby Bobolinks pass through Galápagos because it is on its way to an isolated wintering area outside of the main wintering range. Lévêque et al. (1966) pointed out that small numbers of Bobolinks have been observed in southern Peru during winter, and the Galápagos Islands lie between California and Peru. In 1968, Howell (1975) saw 12 Bobolinks foraging on reeds along a pond's edge in northern Chile in early December. He searched the same place again in 1970 but found no Bobolinks. There are additional undated records from Peru in NatureServe (Ridgely 2002, unpublished data) and in Chile in January (Pettingill Jr., 1983). Finally, it is possible that Bobolinks remain in Galápagos for an extended period, perhaps the entire non‐breeding period. In 2021, while Bobolinks did not use the same sites on San Cristobal Island as documented in Perlut and Renfrew (2016), the same individuals were frequently resighted on a single larger site across a 3‐week period (NGP. unpublished data).

If Bobolinks in Galápagos are not vagrants, what maintains this seemingly inefficient migratory route? The route could be reinforced by conspecific attraction. Like other blackbird species, Bobolinks are highly social. They are considered semi‐colonial during the breeding season, they use social information when prospecting and settling (Nocera et al., 2006), and on non‐breeding grounds, they form flocks and use vocal and visual cues to avoid predation and find foraging areas (Renfrew & Saavedra, 2007). Social information influences young Bobolinks especially, such that individuals will select low‐quality habitats in response to social information over other means of habitat evaluation (Nocera et al., 2006, 2009). Young Bobolinks especially may follow the “wrong” migration route in the process of following erroneous social cues (Betts et al., 2008, found this with respect to selecting breeding habitat). Therefore, one or a few Bobolinks that make an error could have followers. Finally, the route could be maintained to minimize exposure to parasites (Gill et al., 2009; Levin et al., 2016); in this case, Bobolinks avoid continental or island stopovers with known parasite histories but going to the Galápagos prior to ~2000 had no known blood parasites. However, if this migration was maintained to avoid parasites, we are unsure why such a small proportion of the global population uses it, particularly when only 18% of Bobolinks carry *Plasmodium* (Levin et al., 2013). Likewise, we do not yet know if and how parasites, particularly *Plasmodium*, impact Bobolink life histories.

In conclusion, following the proximity hypothesis, our findings provide evidence that the Bobolinks that stopped in Galápagos in 2015 originated from the eastern part of their breeding range, likely in the area in or surrounding Ontario. These findings advance our rapidly evolving understanding of the migratory capabilities of birds, and also raise questions about whether and how seemingly inefficient strategies are maintained over time. Future research is warranted to strengthen and broaden our findings. In particular, genetic data from additional locations in the breeding range, especially in the midwestern and southeastern portions, would help tease apart remaining hypotheses about the alternate migratory pathways. Sampling Bobolinks in Galápagos in additional years would help determine whether migrants consistently originate from the same breeding population, and clarify whether vagrancy is contributing to their occurrence on the Galápagos islands.

## AUTHOR CONTRIBUTIONS

**Noah Perlut:** Conceptualization (equal); funding acquisition (lead); investigation (lead); methodology (equal); project administration (lead); resources (equal); writing – original draft (lead); writing – review and editing (lead). **Logan M. Maxwell:** Formal analysis (equal); investigation (equal); methodology (equal); visualization (lead); writing – original draft (equal); writing – review and editing (equal). **Adrienne Kovach:** Conceptualization (equal); formal analysis (equal); funding acquisition (equal); investigation (equal); methodology (equal); resources (equal); writing – original draft (equal); writing – review and editing (equal). **Patricia Parker:** Conceptualization (equal); writing – original draft (equal); writing – review and editing (equal). **Rosalind B. Renfrew:** Conceptualization (equal); investigation (equal); writing – original draft (equal); writing – review and editing (equal).

## BENEFIT‐SHARING STATEMENT

Benefits from this research accrue from the sharing of our data and results on public databases as described above.

## Supporting information


Appendix S1

## Data Availability

Data will be archived in Dryad (doi: 10.5061/dryad.9s4mw6mmj).
