# Supplementary material for: Breeding origins of a uniquely regular migrant songbird in the Galápagos Islands
Source: Ecol Evol. 2023 Jan 16;13(1):e9697. doi: 10.1002/ece3.9697 (PMC9842894; doi:10.1002/ece3.9697)
Supplement: Supplementary file 1 — Appendix S1 [file ECE3-13-e9697-s001.zip › ECE3_9697_APPENDIX.docx]

Appendix 1: Delta K and Mean LnP(D) plots from STRUCTURE runs (K =1-8) for Bobolink population structure (including seven breeding sampling locations and migrants caught in the Galápagos, compiled in STRUCTURE harvester.


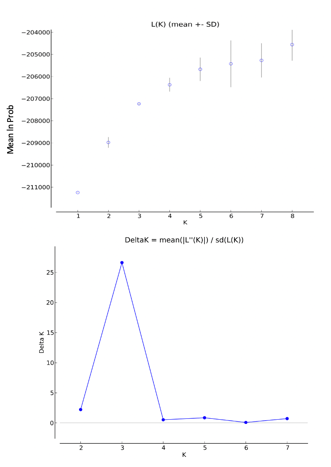


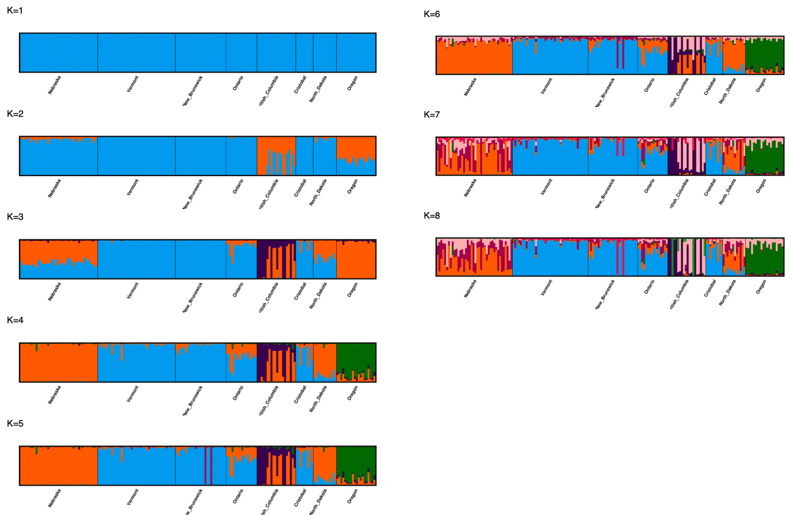
Appendix 2: STRUCTURE bar plots k =1-8 for Bobolink breeding sampling locations (7) and migrants caught in the Galápagos Islands (Cristobal).

Appendix 3: Results of the Monte Carlo cross validation performed in AssignPop to evaluate baseline Bobolink data (seven breeding populations) using neutral SNP panel (3226). Top panel includes assignment accuracy for each sampling location (and overall) across varying proportions of individuals used in the training dataset (0.5, 0.7, and 0.9). Bottom panel includes assignment accuracy for each sampling location using number of individuals in the training dataset (4, 6, and 12).

**
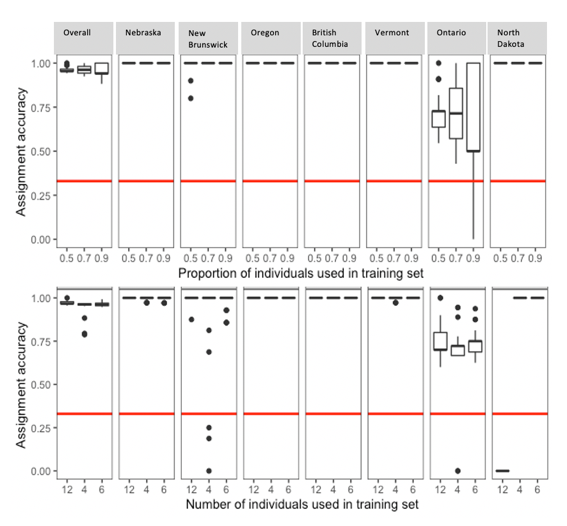
**

Appendix 4: Mixture simulation results from Rubias using the leave-one-out approach with the gene copies method for Bobolinks. In this simulation, breeding sampling location is listed as the reporting unit (unique colors). Top panel includes the average posterior means of group membership across each sampling location with numbers of simulated individuals listed above each. The middle panel plots estimated reporting unit mixing proportions against simulated mixing proportion values, while the bottom plots estimated reporting unit missing proportions against the simulated number of individuals from each reporting unit. A perfectly accurate estimate would fall evenly upon the 1:1 solid line, while any estimates above the line are upwardly biased, and any estimates below the line are downwardly biased.

Appendix 5: Results from the Rubias simulations using Bobolink breeding sampling locations (7) as reporting units and full multilocus genotypes as the method. Top panel includes the average posterior means of group membership across each sampling location with numbers of simulated individuals listed above each. The middle panel plots estimated reporting unit mixing proportions against simulated mixing proportion values, while the bottom plots estimated reporting unit missing proportions against the simulated number of individuals from each reporting unit.

Appendix 6: Results from the Rubias simulations using Bobolink regional population groupings as reporting units (unique colors) and full multilocus genotypes as the resampling method. Top panel includes the average posterior means of group membership across each sampling location with numbers of simulated individuals listed above each. The middle panel plots estimated reporting unit mixing proportions against simulated mixing proportion values, while the bottom plots estimated reporting unit missing proportions against the simulated number of individuals from each reporting unit.

Appendix 7: Results from the Rubias simulations using regional population groupings as reporting units (unique colors) and gene copies as the resampling method. Top panel includes the average posterior means of group membership across each sampling location with numbers of simulated individuals listed above each. The middle panel plots estimated reporting unit mixing proportions against simulated mixing proportion values, while the bottom plots estimated reporting unit missing proportions against the simulated number of individuals from each reporting unit.


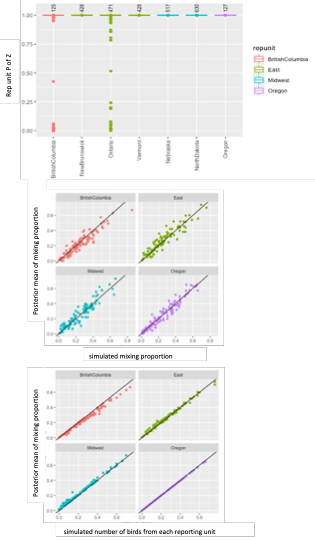


Appendix Table 1: Results from the mixture analysis performed in Rubias, using the seven Bobolink breeding sampling locations as the reporting unit and without the parametric bootstrapping correction.

| **Individual** | **Rep unit** | **P of Z** |
| --- | --- | --- |
| Galápagos 44619 | Ontario | 1.000 |
| Galápagos 44620 | Ontario | 1.000 |
| Galápagos 44621 | Nebraska | 0.999 |
| Galápagos 44622 | Ontario | 1.000 |
| Galápagos 44623 | Ontario | 0.919 |
| Galápagos 44624 | Ontario | 1.00 |
| Galápagos 44625 | Nebraska | 0.999 |
| Galápagos 44626 | Ontario | 1.00 |
| Galápagos 44627 | Nebraska | 1.00 |
