# Supplementary figures and images for: Breeding origins of a uniquely regular migrant songbird in the Galápagos Islands
Source: Ecol Evol. 2023 Jan 16;13(1):e9697. doi: 10.1002/ece3.9697 (PMC9842894; doi:10.1002/ece3.9697)

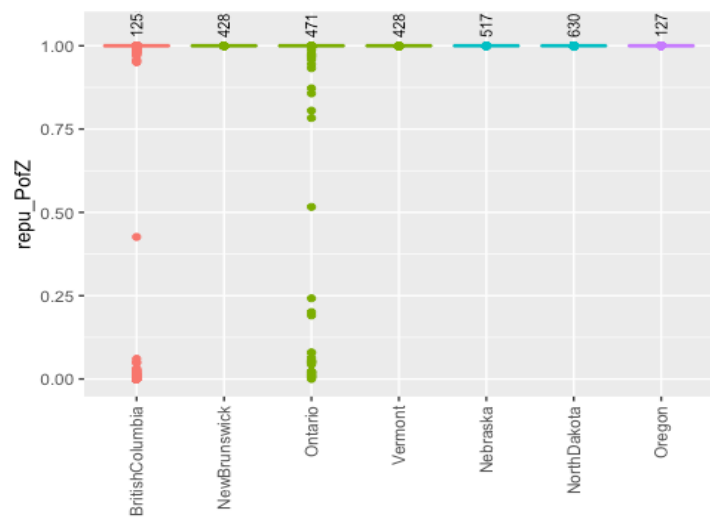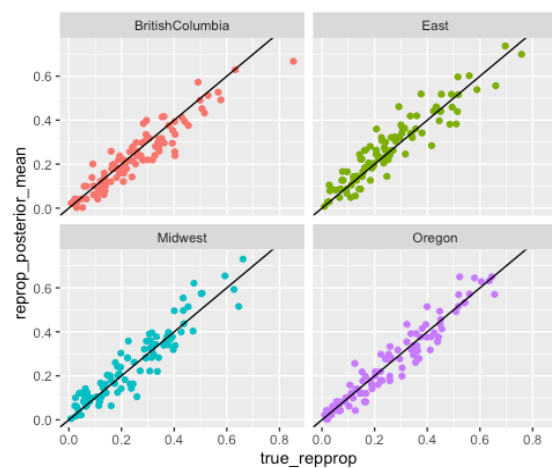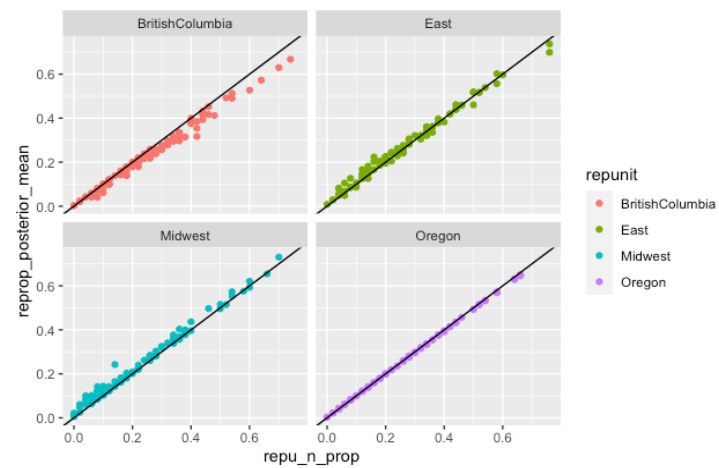

Supplement: Supplementary file 1 — Appendix S1 [file ECE3-13-e9697-s001.zip › ECE3_9697_SupFig6.pdf]

K=1

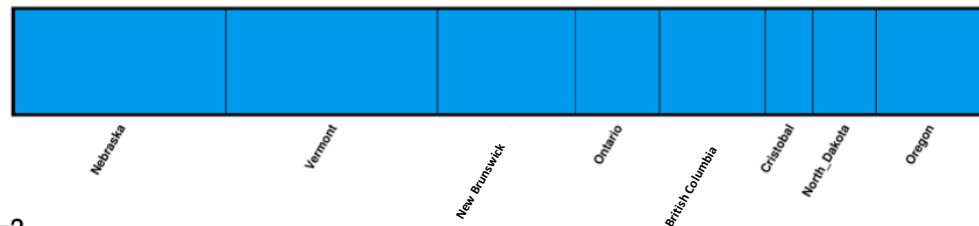

K=2

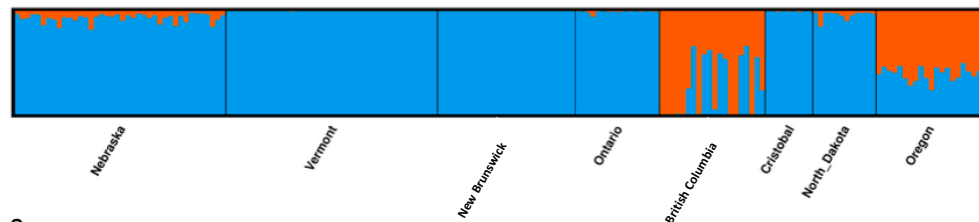

K=3

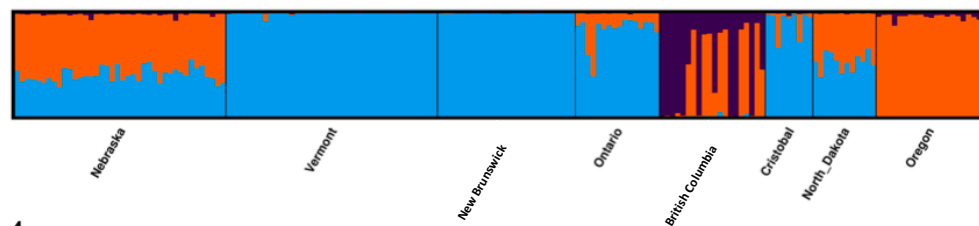

K=4

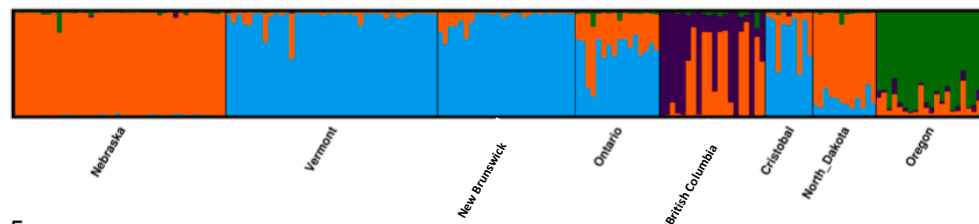

K=5

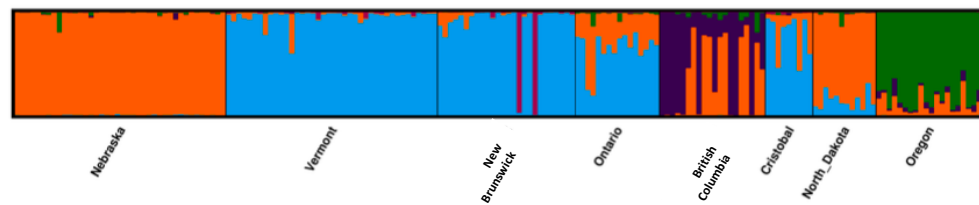

K=6

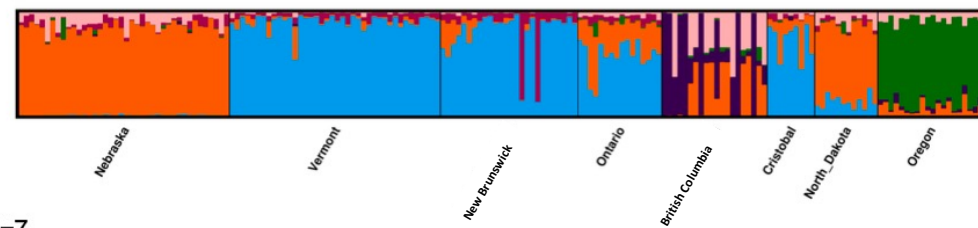

K=7

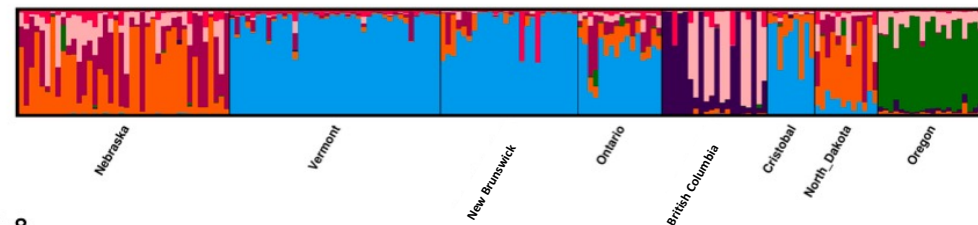

K=8

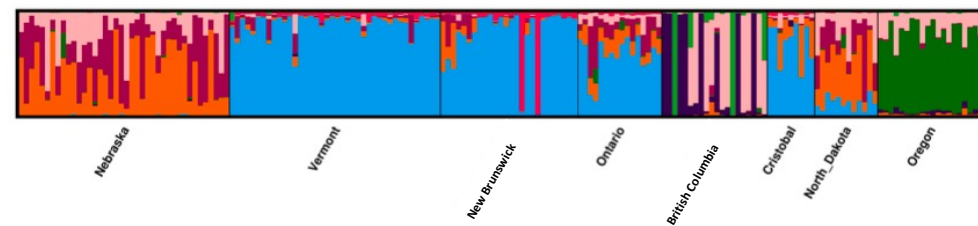

Supplement: Supplementary file 1 — Appendix S1 [file ECE3-13-e9697-s001.zip › ECE3_9697_SuppFig2.pdf]

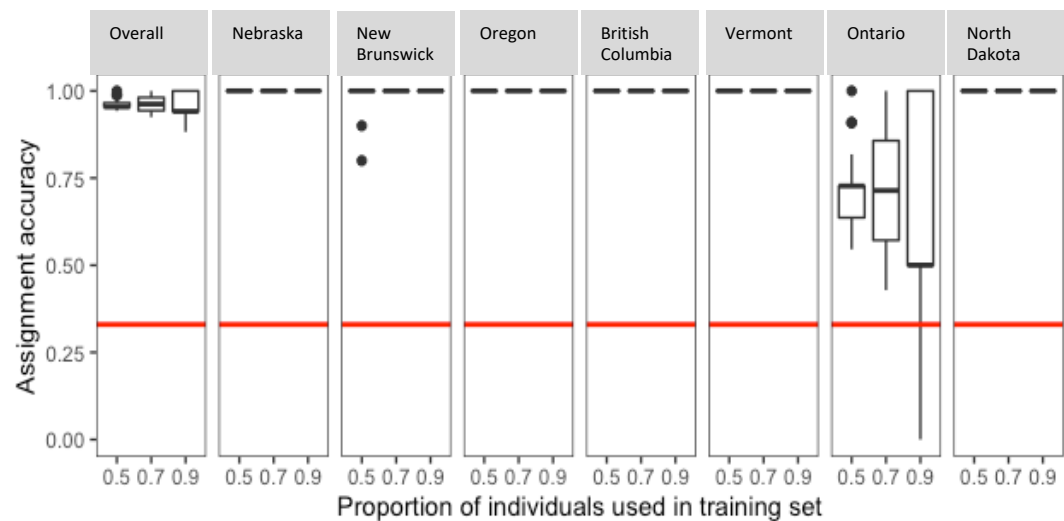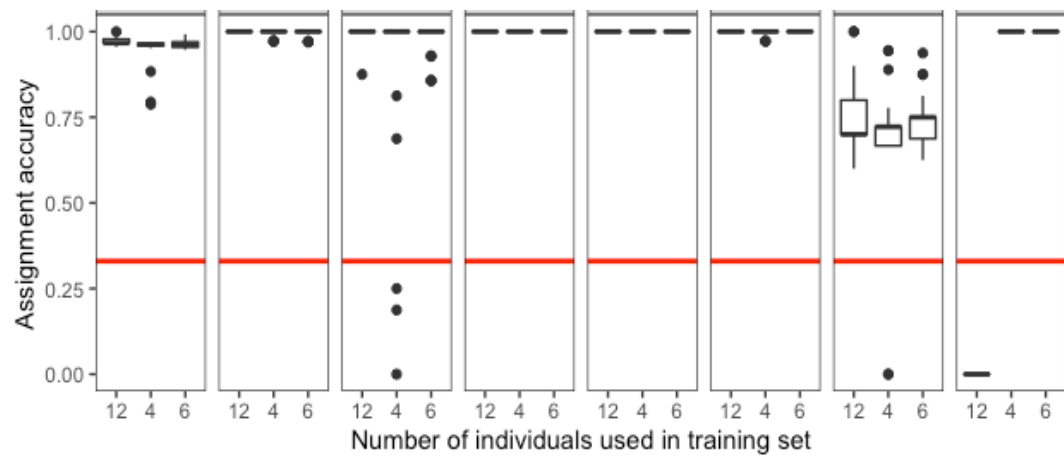

Supplement: Supplementary file 1 — Appendix S1 [file ECE3-13-e9697-s001.zip › ECE3_9697_SuppFig3.pdf]

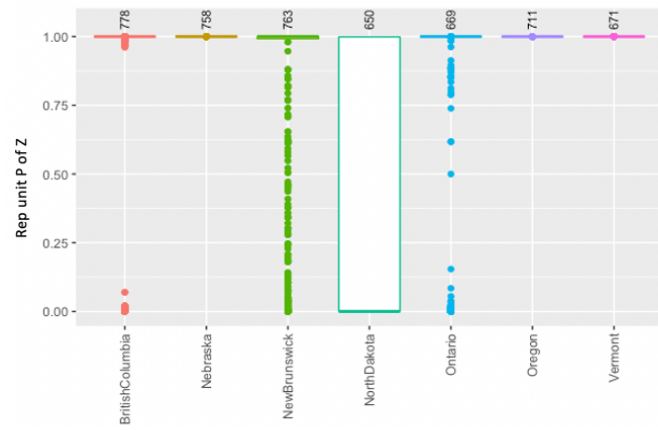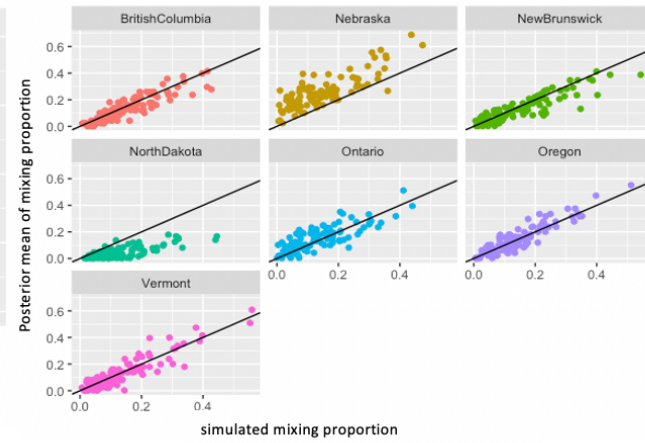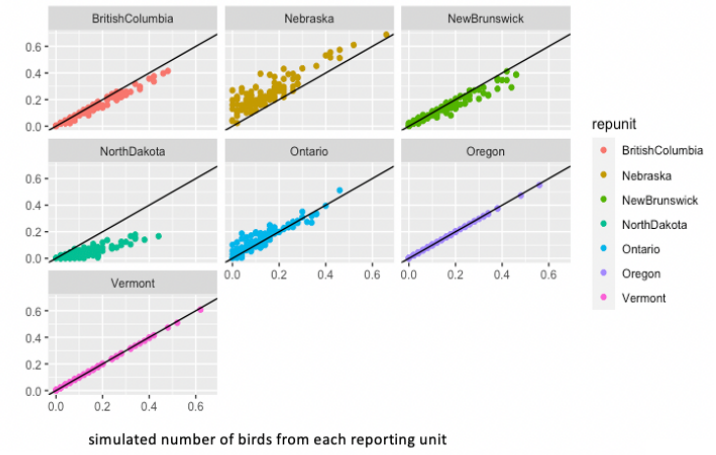

Supplement: Supplementary file 1 — Appendix S1 [file ECE3-13-e9697-s001.zip › ECE3_9697_SuppFig4.pdf]

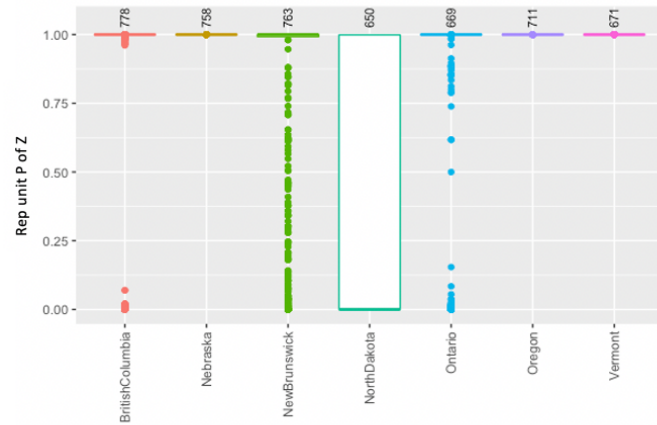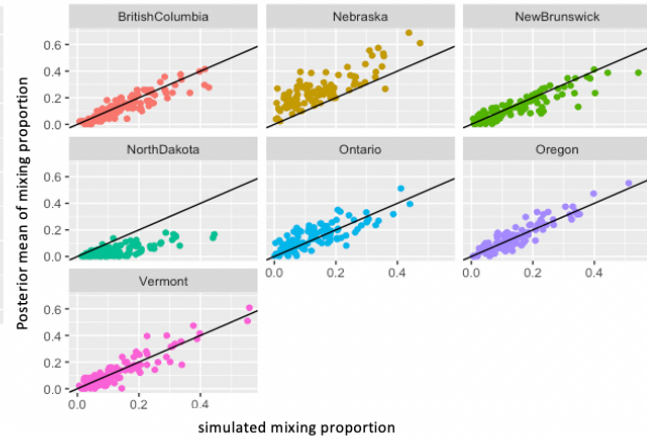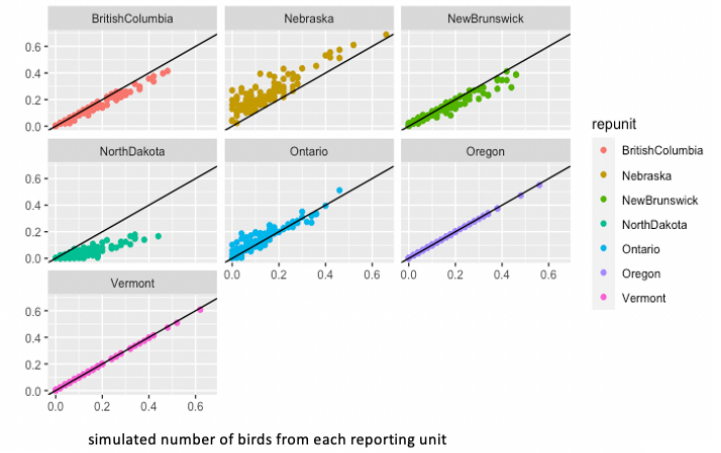

Supplement: Supplementary file 1 — Appendix S1 [file ECE3-13-e9697-s001.zip › ECE3_9697_SuppFig5.pdf]

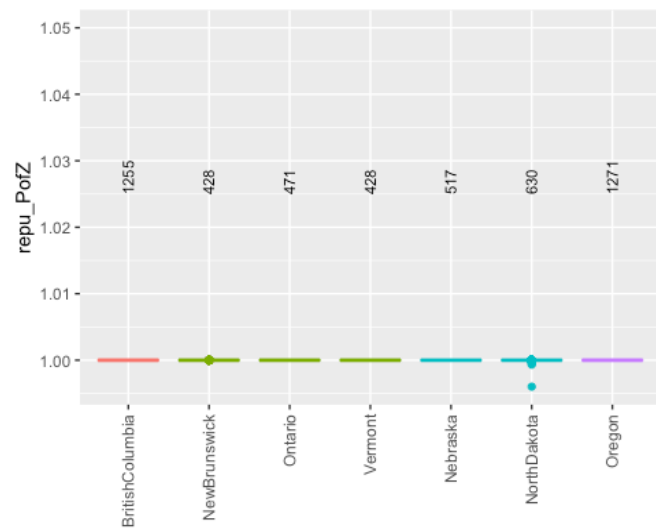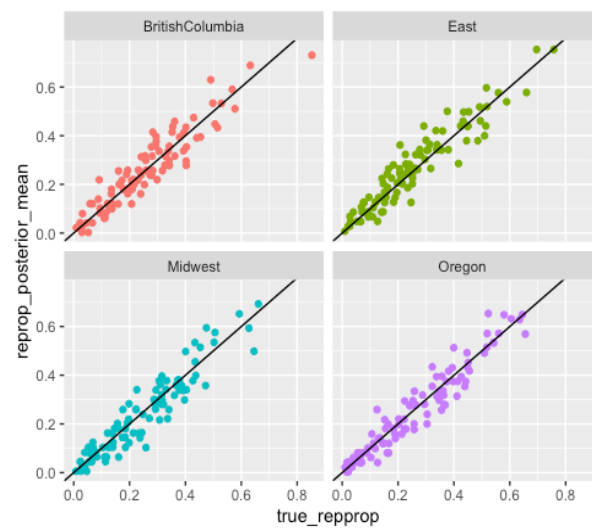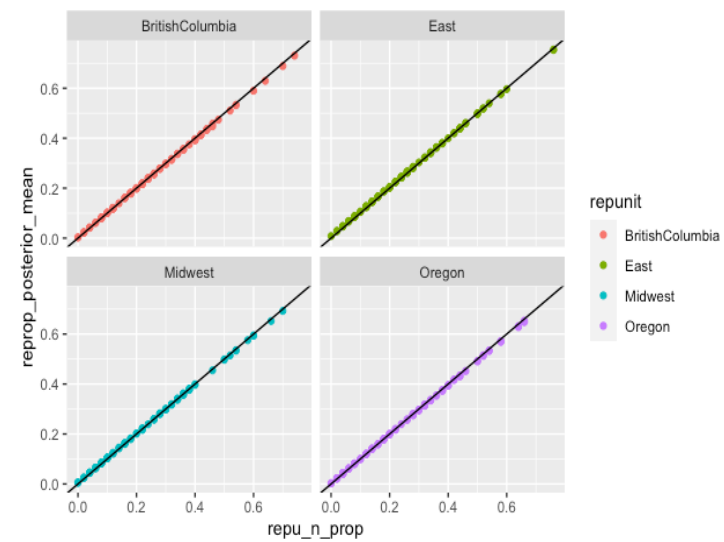

Supplement: Supplementary file 1 — Appendix S1 [file ECE3-13-e9697-s001.zip › ECE3_9697_SuppFig7.pdf]

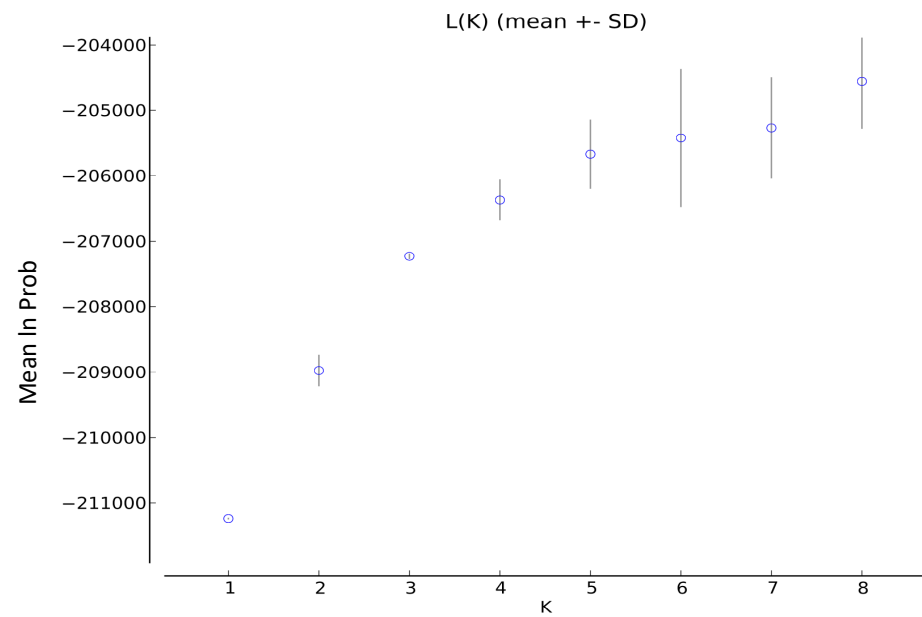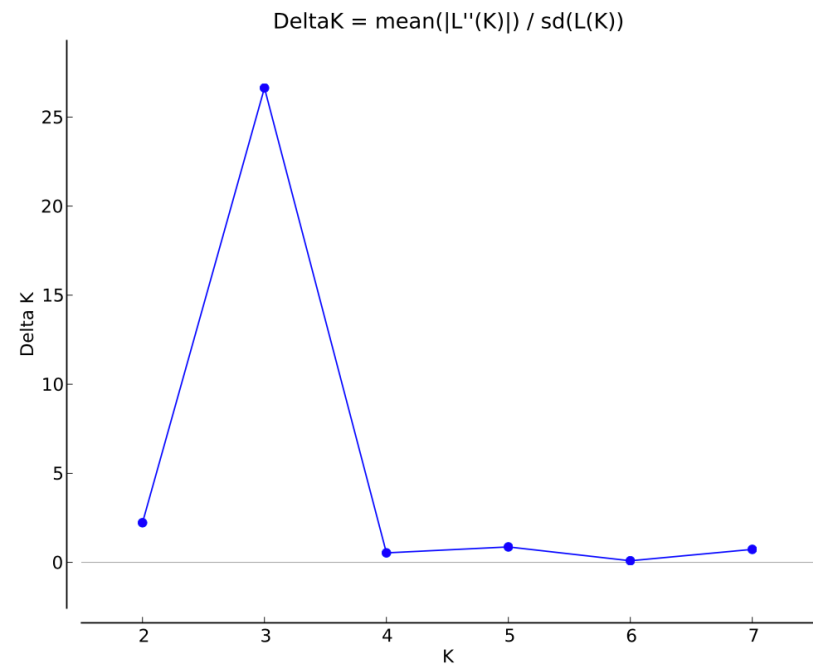

Supplement: Supplementary file 1 — Appendix S1 [file ECE3-13-e9697-s001.zip › ECE3_9697_SuppFigure1.pdf]
